# Peritoneal Inflammation

**Published:** 1820-09-01

**Authors:** 


### Medico-Chirurgical Review,

AND

JOURNAL OF MEDICAL SCIENCE

( Analytical Series )

Nec tibi quid liceat sed quid fecisse decebit
Occurrat mentemque domat respectus honesti. CLAUD.

Vol. I.] SEPTEMBER 1, 1820. [No. 2.

### I.

### PERITONEAL INFLAMMATION.

1. *Histoire des Phlegmasies, ou Inflammations Chroniques.* Par F. J. V. BROUSSAIS, M. D. &c. Chapitre iv. De L'Inflammation du Peritoine. (Vol. II.)

2. *Peritonite :—Inflammation du Péritoine.* Par M. M. GASC.

[Dictionnaire des Sciences Medicales, Vol. XL.]

3. *Researches on the Pathology of the Intestinal Canal.* By JOHN ABERCROMBIE, M. D. &c. &c.

[ Edinburgh Medical and Surgical Journal, No. LXIII.]

4. *A Practical Treatise on various Diseases of the Abdominal Viscera.* By C. R. PEMBERTON, M. D. &c. &c. &c. 4th Edition, revised and corrected. London, 1820.

5. *Effets de l'Inflammation sur le Peritoine, reconnus par les Ouvertures de Cadavres.* Par M. M. MONTFALCON, M.D. &c. &c.

[Dict. des Sciences Med. tom. 40.]

IT is in vain to look for *minute* pathological anatomy beyond the circle of the last twenty years. Candour also obliges us to confess that (with a very few honourable exceptions)* it is not in this country we need look for it at all.

* Particularly Dr. Baillie's works.

This statement will highly offend the national pride and prejudice of those whose researches are limited to their own case-books, and who cannot conceive that medical superiority may exist beyond an arm of the sea, or a fresh-water stream. We believe we have already exhibited proofs that our continental brethren have out-stripped us in pathology, and we shall steadily persevere in pourtraying the salutary examples of unwearied industry which they have set us, however bitter to swallow, or hard to digest, the potation may be.

No practitioner is unaware of the great importance and danger of abdominal inflammation; but even to this day, inflammations of the different tissues are too often confounded together, without due regard to their anatomical, physiological, and pathological peculiarities. How common is it to confound inflammation of the peritoneum with that of the organs which it covers, and to talk of isolated affections of the stomach, intestines, womb, &c. under the designations of gastritis, enteritis, hysteritis, as though the inflammation attacked, at one and the same time, the whole of the tissues composing those viscera! Yet nothing is more certain than that almost every acute inflammation of the organs above-mentioned, begins in a single tissue of their structure, with corresponding symptoms, and spreads thence to other tissues, with more or less rapidity, according to the violence of the disease, and the mode of treatment, accompanied also by a train of phenomena indicative of the new structures successively invaded.

In by far the greater number of those cases termed gastritis and enteritis, the inflammation is pure peritonitis at the beginning, and the disease takes its name improperly from the organ over which the principal inflamed portion of peritoneum is spread;* though, in such instances, we rarely or never find the inflammation bounded by the limits of a single viscus, but wandering undefined over a greater or less extent of the peritoneal tunic. Every one now acknowledges that the pleura is frequently inflamed where the parenchymatous structure of the lungs escapes; and it is precisely so with the peritoneum, excepting that in the latter there is more danger than in the former. There is, indeed, a very close analogy between pleuritis and peritonitis. They begin, like all other

---

* This observation of Broussais seems confirmed by the later writings of Dr. Abercrombie. "Inflammation of the peritoneum, says Dr. A. may occur in a more limited form than in the disease which I have now described, and, according to the seat of it, *may assume the appearance* of disease of various organs, as the bladder, the kidney, the liver, &c." 167.

acute diseases, with chilliness, succeeded by heat, fever, &c. They show the peculiar character of *serous* inflammations—extremely acute pain, wandering or fixed, accompanied by a distressing sense of internal heat. As in pleurisy the pain is complained of in a single point, although the whole sheet of pleura be inflamed; so, in peritonitis, the pain or tenderness is generally referred to a particular part, even when the phlogosis is very extensive.

"As cough and expectoration (says M. Gasc) take place in pleurisy, so nausea and vomiting are common attendants on peritonitis. As pleurisy is under atmospheric influence, and sometimes appears to spread epidemically, so does peritoneal inflammation. As hydrothorax frequently results from pleuritis, so does ascites from peritoneal inflammation. Pleurisy sometimes terminates in suppuration, and produces empyema; peritonitis occasionally gives rise to a puriform effusion that almost invariably proves mortal. In pleuritic inflammation, coagulable lymph is thrown out, and adhesions are formed; in peritoneal inflammation, the intestines are often glued together by the same substance." *Dict. des Sciences Med. tom.* 40, *p.* 497.

After these preliminary observations, we shall proceed systematically in drawing up a synthetical article on the important disease now under review.

### I. Etiology.

The *predisposing* causes of peritoneal inflammation are involved in obscurity, and may depend much on idiosyncrasy. Among these, however, have been ranged, plethora, irritable habit, abuse of inebriating liquors, ultra-excitement of the passions, cold seasons of the year, damp habitations.

The *exciting* causes are various; some are mechanical, some chemical. Thus, compression of the abdominal viscera, blows or falls on that region; in short, every contusion or general commotion of the abdomen may determine a focus of irritation, and ultimately inflammation on its peritoneal lining. Mechanical irritation also results frequently from internal friction or pressure, as of the gravid uterus, extra-uterine conceptions, enlarged ovaries, or other morbid growths within the abdomen. M. Broussais considers violent and long-continued corporeal exertions, violent and repeated contractions of the abdominal muscles in vomiting, the centripetal oscillations of the blood in the cold stages of intermittents, and strictures of the colon or rectum producing unnatural contortions and friction of the intestines on one another, as causes of peritoneal inflammation.

Among the chemical internal irritations may be placed all extravasations not quickly absorbed, as blood, bile, chyle, urine, fæces, and perhaps the morbid serous secretions of the tissue itself. But the grand causes are to be sought in the action of a cold atmosphere on the surface of the body, the application of wet or cold, especially to the feet, when the person is in a state of corporeal inaction, the neglect of changing wet clothes, the drinking of cold liquids when the body is heated, and whatever suddenly interrupts certain functions of the system, as the suppression of perspiration, the lochia, and the menstrual discharge.

Over the causes which produce epidemic dispensations of this disease, the same veil of mystery hangs, that conceals from our view the Etiology of other Epidemics. M. Broussais saw the disease epidemic, and apparently contagious among the French armies, in various parts of the continent—in Germany, Holland, and Italy. We have all seen puerperal peritonitis epidemic in our own country. The translation of rheumatic, arthritic, or erysipelatous inflammations from the joints or surface of the body to the interior tissues, is not to be overlooked by the observant practitioner, or ridiculed by the sceptical sciolist.

According to the force of the cause and the constitution of the subject, we have peritoneal inflammation of the acute or chronic form. These forms may not be very distinct, when they are passing to or from one another; but, at all other times, they are sufficiently distinguishable, and accompanied by marked peculiarities and features.

### II. Symptomatology.

*Acute Peritonitis* is generally ushered in with horripilatio or cold chills, sometimes amounting to rigors, accompanied by malaise and weariness of the limbs. It not unfrequently happens, that this chilly stage continues for even one, two, or three days, before re-action takes place; at other times, however, this cold stage is of short duration; and heat succeeds, more or less pungent, with head-ache, constriction of the epigastric region, and frequent, hard, concentrated pulse. The face is usually pale, and often covered with cold clammy sweat; but the abdominal pains produce certain muscular contractions of the face, which give the countenance a peculiar sharpness of feature, characteristic of this disease. Sometimes there is a degree of livor of countenance; at others, it is animated with an air of agitation; or fixed, and determined. The intellectual faculties are generally performed soundly till the last, with occasional delirious wanderings, and convulsive movements of the head and limbs. To the foregoing symptoms we may add sleeplessness, thirst, a sense of stupor, with coldness of the extremities. Little can be drawn from the state of the secretory and excretory functions, which vary much, according to the intensity of the disease, and other circumstances of the patient. Such are the *general* phenomena; the *local*, or characteristic symptoms, deserve a separate consideration.

Peritonitis being declared, the abdomen becomes painful, sometimes to such a degree, that the least pressure is insupportable, and even the weight of the bed-clothes is distressing. This pain is very different from that attending dysentery, being infinitely more acute, and constant. It also differs from that attending phlogosis of any particular abdominal organ. In peritonitis the pain is sometimes fixed, and then it appears confined to a single point; at other times, it is general or wandering, according to the extent of the inflammation. The patient dare not lie in any other position than on the back, and is afraid of moving, on account of the exasperation of pain attending every motion. Both tension and swelling may now be detected in the abdomen, partly from flatulence in the bowels, partly from emphysema of the subjacent cellular substance. Hiccup, nausea, and vomiting supervene, with anxiety, frequent and laborious respiration, especially inspiration, from the descent of the diaphragm on the abdominal viscera. There is sometimes diarrhœa, but generally constipation of the bowels.*

Such are the open forms of the disease. But the symptoms above enumerated are often modified by age, constitution, season, and other circumstances. Thus, in some subjects, of feeble powers and advanced in age, peritonitis will creep on without fever; nay, without local pain—and yet the disorder will go on to complete disorganization of the peritoneum, or such an effusion of serous fluid as gives the complaint the character of ascites. In some subjects, the thickness of the muscles and parietes of the abdomen renders the pain of pressure scarce perceptible. In such case, M. Broussais directs us to make *lateral* pressure towards the centre of the abdomen.

"Elle (douleur) était plus difficile à supporter quand on la faisait (pression) lateralement, en la derigeant vers le centre. Ce signe est un des meilleurs pour faire découvrir les péritonites obscures." *Vol. II. p.* 413.

---

* "Elle arrête (says Broussais) les excretions alvines On concoit qu'elles sont impossibles, lorsqu'on remarque que tous efforts pour all[illegible]r à la selle ou pour uriner, ainsi que les secousses de la touse, et de l'éternument, sont insupportable." *Vol. II. p.* 492.

To this symptomatology of the French authors we can add little from our countryman, Dr. Abercrombie. Indeed, if the reader will compare Dr. A's description of inflammation of the peritoneal coat of the intestines, in pages 666 and 667 of the Edinburgh Journal, he will find it, in many places, almost a literal transcript from Broussais, published twelve years ago. By this, we do not mean to say that Dr. A. copied from Broussais; but we think that he ought to have extended his *researches* a little farther, and noticed the investigations of so distinguished a pathologist as M. Broussais. These remarks will apply still more forcibly to the pathology than to the symptomatology of the disease under consideration.

In some epistolary communications, which the writer of this article lately had with an esteemed friend and able physician,* there are some passages which are highly worthy of notice.

"Though we chiefly judge (says Dr. D.) of the degree of abdominal inflammation by the severity and constancy of the *pain;* yet a fatal disease may be going on, though the pain be far from acute, or only recurring at intervals. The grand criterion is the ability of bearing manual pressure; and *this* we are not to estimate solely by the complaint, but by the countenance also of the patient. Even gentle pressure causes a sudden retraction of the lips, and expression of pain, as if he were pierced by a sharp instrument. On the other hand, we are not to overrate the tenderness, (though it is the surest diagnostic) by the wincing of the patient, especially if very irritable, or young, and afraid of being hurt—or if the bowels happen to be uneasy and distended with flatus at the time. The pulse is deceitful, and only to be regarded in conjunction with the other phenomena. Though the pain, tenderness, and other symptoms have subsided, yet this abatement is not to be relied on, so long as the pulse keeps up, particularly if the tongue does not clean, or after being so, is disposed to resume its coat; in such circumstances, there is great danger of relapse, from any dietetic indulgence, exposure to cold, or getting up too soon. Bearing the erect posture well, is certainly a proof of diminished tenderness; but the attempt should not be hazarded too early. When the pain returns at intervals, but is not of long duration, it is often difficult, particularly in young subjects, to know whether it is from unsubdued inflammatory action, or to be referred to griping colicky pains from the operation of medicine. Where we are in doubt whether pain be inflammatory or spasmodic, the safe plan is to treat it as the former. The bowels are generally obstinately closed during the first days of enteritis, with vomiting in proportion to the degree of anti-peristaltic motion, sympathy, or

* Dr. Dickson of Clifton.

the extent, and spreading upwards of the inflammation. Even after free motions have been procured, constipation is very apt to recur, and the bowels are tardy and difficult to manage throughout the whole course of the disease."

This observation of Dr. Dickson accords with our own experience, and with that of all the best authors and practitioners. We cannot but regard the following sentence of Dr. Abercrombie, therefore, as tending to impress the inexperienced mind with a description which is, generally speaking, erroneous, and a principle which is exceedingly dangerous.

"We have seen (says Dr. A.) the bowels obstructed; we have seen them natural; and we have seen them spontaneously loose: and under these varying circumstances, we have found the disease going on with equal certainty, and equal rapidity, to a fatal termination." *Ed. Journ.*

Those who have not seen much of peritoneal or abdominal inflammation, would naturally conclude that the above states were so nearly equal in point of occurrence, that nothing was to be thence inferred; whereas we appeal to the observation of practitioners, whether constipation is not as general a concomitant of *peritoneal* inflammation, as dysenteric purging is of inflammation of the mucous membrane of the intestines. Exceptions will occur to all general rules, of course; but really Dr. Abercrombie appears to us to have confounded in the above sentence, two very different inflammations—that of the internal and that of the external coats of the intestinal canal—requiring a very considerable modification of treatment. This error would be of less consequence, did it emanate from a weaker authority. As it stands, it is calculated to do harm; and hence our anxiety to combat it.

We may here cite the authority (no mean one by the bye) of Dr. Gregory, the present professor in Edinburgh.

"Enteritis (says he) is generally attended with costiveness; *when* the belly is open, it is a good sign." *M.S. Lectures.*

"Bleeding (says the same Professor) is particularly necessary; but the great view is to keep the belly open. Whenever it is in a natural state, the danger is over."

Dr. Gregory warmly recommends laxative glysters, as superior to purgatives, by the mouth.

Dr. Pemberton's authority may be also quoted for constipation being a general attendant on enteritis.

"The bowels (says this distinguished Physician) are obstinately costive; what is injected is returned unaltered; and what passes from the rectum unsolicited, is devoid of the ordinary smell and colour of fæces." *4th. Edit. p.* 177.

The march of acute peritonitis is generally short.

"I have never (says Broussais) seen peritoneal inflammation, *accompanied by much pain and fever*, prolonged beyond the medium duration of inflammations of other serous structures—from ten to twenty days. I have remarked, that when the disease did not give way in that space of time to proper treatment, it proved fatal suddenly. I have never seen this inflammation pass from a state of violence to that of tranquillity, after going through the acute stadia, as we sometimes observe in inflammations of the chest, and of the mucous membrane of the bowels. Of the chronic cases of peritonitis which I have met, a few had pain and fever for about three or four days, at the utmost; but the majority of cases had crept on so insensibly, that no account could be obtained of the commencement of the inflammation." *Vol. ii.*

This inflammation may terminate by resolution, suppuration or effusion, gangrene, and chronic peritonitis.

*Resolution.* The termination by resolution generally takes place between the fifth and tenth day; sometimes the fifteenth or twentieth. It is announced by cessation of pain, fever, and other inflammatory symptoms; a re-establishment of function in the contiguous organs; a power of turning to one or both sides in bed; a disappearance of the nausea and vomiting; free evacuations from the bowels, kidnies, and skin; refreshing sleep.

*Suppuration and Effusion.* This is a frequent termination in fatal cases; and it is difficult to ascertain the precise period when pus or effusion is produced. There is generally a diminution of the abdominal pain, with a sense of weight and oppression; irregular chills; a softness in the pulse; paleness of the countenance; coldness of the extremities—death.

In peritoneal suppuration we have no ulceration or other breach of continuity in the membrane. The purulent matter apears to be thrown out by a kind of exhalation or secretion, as the ordinary serous fluids. The colour and consistence of the purulent matter are very various; being reddish, greenish, or white, like milk, with shreds of membrane, and albuminous flocculi floating in it.

The internal surface of the peritoneum in health, is always secreting a serous halitus. When inflamed, the gorged state of its vessels will sometimes cause a sanguineous effusion, instances of which are related by Broussais. But more commonly, in peritoneal inflammation, there is a preternatural serous secretion, of various colours and consistencies, which accumulating in the abdomen, increases the irritation and aggravates the inflammation.

The absence of redness in the peritoneum, on dissection, is no proof that phlogosis did not exist previously; for Bichât has well observed, that death having removed all irritation form this membrane, its capillary vessels may so far shrink, in some cases, that no turgescence or redness is visible, in the same manner as erysipelas, morbilli, scarlatina, &c. disappear from the surface of the body after death.

*Gangrene.* Of all inflammations of *serous* membranes, peritonitis, when violent, is most disposed to run into gangrene. The phenomena indicative of this termination are sufficiently obvious. A sudden cessation of the abdominal pain; smallness of the pulse, which becomes concentrated and intermitting; prostration of strength; and facies hippocratica, announce the approach of death.

Another termination of acute peritonitis, is so important that it must occupy a considerable share of our attention.

Chronic Peritonitis. When acute inflammation of the peritoneum continues beyond the fifteenth or twentieth day, it becomes chronic; but this last form of the disease is by no means always preceded by acute peritonitis. It often steals on, from the same causes as were before enumerated, in a slow and insensible manner; especially when the patient is advanced in age, or debilitated in constitution. Particular occupations, which cause pressure on the abdomen, predispose to, if they do not excite this disease; and, in its etiology, may be ranked unhealthy localities; cold and moisture; poverty; prolonged residence in hospitals; fatigue; forced marches among the soldiery; unwholesome food acting as an irritation on the abdominal viscera; protracted intermittents; every kind of slow effusion into the cavity of the peritoneum.

Its approach is so insidious, (when it does not succeed the acute form) that we never see it at its commencement; consequently, we can only describe its early symptoms by watching the transition from the acute to the chronic forms of the disease. The patient experiences a kind of constant morbid feeling in the abdomen, rendered more obvious by pressure. It is the property, however, of *chronic* inflammations of the serous membranes to excite very little acute pain, "being rather acute tenderness than actual pain."—*Abercrombie.*

Baglivi, Morgagni, and others have seen the pleura, a membrane so sensible in acute pleuritis, completely disorganized, and even profoundly suppurated, in chronic inflammation, without the patient having ever complained of the least pain. It is, therefore, highly imperative on us to watch the patient after an attack of acute peritoneal inflammation, and not be thrown off our guard by the cessation of suffering. Sometimes, however, when chronic succeeds acute peritonitis, the patient complains of a fixed pain, like a stitch, at the epigastrium, which is aggravated by any sudden movements, unusual exercise, sneezing, &c.

If we examine the abdomen, a slight tumefaction, and glossiness of the surface will be perceived, especially towards evening. Dr. Pemberton observes, that the patient—

"Only complains, after fatigue, of a certain degree of tightness, and pricking soreness across the abdomen, from one os ilium to the other. This state will continue, with little variation, for many months; during which time the operations of the bowels will sometimes proceed naturally, though more commonly the patient is costive. There is no tension of the skin of the abdomen, as in the acute species: on the contrary, I have more than once observed the skin and abdominal muscles to sit loosely upon the peritoneum, which has given a sensation to the touch, as of a tight bandage underneath, over which the skin and muscles may be said (as it were) to slide. The patient always complains more of tightness than of pain; and as this tightness is much increased by any congestion in the bowels, the relief which he experiences from evacuating their contents, leads him to attribute his sensations to an habitual costiveness." *Fourth Edition, p.* 11.

The appetite is often preserved, and the digestion but little deranged; in which cases, we may conclude, that the peritoneum reflected over the stomach, is not very deeply involved in the disease. At other times, however, there is vomiting, but this is not to be considered a pathognomonic symptom of chronic peritonitis. Broussais mentions a sensation, as though a ball were rolling about in the abdomen, and sometimes approaching the throat; this he attributes to the agglutination of the intestines, which form, with the gorged mesenteric glands, a round and mobile mass in the belly, often without any effused fluid.

The general, or symptomatic phenomena of chronic peritonitis are very obscure. The pulse is natural, except towards evening, when it becomes quickened, with some augmentation of heat, a little colour on the cheeks; and some dyspnœa and cough, when the patient lies horizontally.* These symptoms give cause to apprehend, that some effusion is going on in the abdomen, and the suspicion is strength-

* Dr. Pemberton says, he has not observed these evening accessions of febrile symptoms; but rather a languor, with a pallid, and doughy appearance of the face.

ened by the appearance of any œdema of the ancles, and paucity of urine. Constipation of the bowels is not so common in the chronic, as in the acute peritonitis. Diarrhœa occasionally takes place towards the close of the patient's life.

The *Diagnosis* is sometimes extremely obscure in this disease. When the case is unequivocal, the prognosis is almost invariably unfavourable. In short, "when the disease has made any progress, says M. Gasc, it is rare that we have not *tubercular depositions*, more or less abundant, in the peritoneum, and the matter of which they are formed, being little susceptible of absorption, is, in itself, ultimately, the cause of death."

"En effet, dans la maladie parvenue à ce degré, il est rare qu'il n'yait pas, dans le tissu du peritoine, des *depots tuberculeux* plus ou moins abondans, et la matiere qui la forme, étant peu susceptible d'etre absorbée, serait, à elle seule, à la longue, une cause de mort."

Our readers will easily see, that the French authors, under Review, are describing evidently Dr. Baron's "tuberculated accretions of the serous membranes;" with this difference, that they place the disease to the account of chronic inflammation of the peritoneum—he to an hydatid origin, independent of inflammation. Time must ultimately decide between them. In the mean while it is necessary, in obedience to the good old maxim, "suum cuique," to state, that neither the French nor the English authors have the merit of originality in ascribing the origin of tubercles to hydatids. That sagacious pathologist, MORGAGNI, has distinctly insisted on this identity, in several places; but the following instances are sufficient for our purpose.

"The observations which I have very frequently made upon the tunica albuginea and vaginalis of the testis, induce me to believe, that the membraneous laminæ of the hydatids, or of the coats in which they are formed, after they have, by rupture, poured out their fluid contents, first contract themselves and their vessels into the form of a caruncle; and, unless a fresh fluid continue to flow thither, are finally so indurated, and dried up, *as to represent those white and hard tubercles of a roundish figure, some larger in their size, and some less, as the hydatids had been, with which the internal surface of the peritoneum, and the production of it over the external surface of the spleen and intestines were beset.* Postquam disruptæ hunc effuderunt, se suaque vascula in carunculæ forman primum contrahere, et nisi novus illac humor effluere pergat, indurari, et exsiccari denique sic, ut alba illa, et dura subrotunda tubercula representent alia aliis, ut hydatides fuerant, majora aut minora, qualibus in descripta virgine intima peretonei facies, ejusque productio, &c." *Epistle* 38, *Article* 35.

Again, in the same Epistle, Morgagni observes—

"Finally, read over again what I have formerly written to you,* of hard granules, or tubercles being prominent on the internal surface of the peritoneum and pleura, and you will certainly find *the series of changes that I have described.* It happened some years ago, that, in a woman who had been taken off by an ascites, the external coat of the intestine was found to be distinguished with very frequent tubercles. When I first examined them, they resembled small turgid lenticular glands; but they were without an orifice, and solid, and seemed to be made up neither of glandular, nor of a fleshy substance; but to be of a middle nature, as it were, betwixt both. I judged that I could determine on nothing more probable, in regard to them, than to suppose, that they were the *remains of ruptured hydatids contracted into themselves.* Censui nihil veri similius a me posse statui, quam si disruptarum hydatidum reliquias esse conjicerem, in se contractas, ut siccæ esse possent, et duræ."

The above, and many other passages prove, that Morgagni maintained the hydatid origin of tubercles; but this author does not assent to the doctrine of Wharton and others, that these hydatids were formed by certain "interstices of the lymphæ-ducts, for (says he) it is long ago that Ruysh admonished us of a great number of hydatids being found in the placenta uteri; sometimes, as I have also seen, and in other parts, in like manner, wherein no lymphatics are found." *Epistle* 38, *Article* 38.

If Morgagni then has anticipated our countryman, he is, at the same time, a strong authority in favour of Dr. Baron's theory.

The duration of chronic peritonitis is extremely variable—it may destroy the patient in a few months; and he may linger for years under it. Twelve years ago, M. Broussais pronounced it incurable. Since that time, however, more extensive experience has presented to this able physician many instances of recovery.

### III. Morbid Appearances in Acute Peritonitis.

The results of numerous dissections have long proved, that the peritoneum may be partially or totally inflamed, without any similar affection of the subjacent organs. In numerous instances the muscular and mucous tissues of the

* Epistle 16, No. 30; and Epistle 22, No. 186.

stomach, intestines, and other hollow viscera, were found perfectly sound, even when the peritoneal coat was gangrened. In general, the traces of inflammation are more and more strongly marked, according to the duration of the disease, and its degree of violence. Sometimes the vessels of the peritoneum are seen finely injected; at others, the redness is scarcely perceptible, owing to the reflux of blood from the capillaries, in articulo mortis.

Bayle, Broussais, and others have noted the following morbid appearances after *acute* peritonitis. 1mo. Redness, thickenings, and even eschars, which penetrated to the mucous membrane of the peritoneum. 2do. Solid exudations, in form of false membranes, lining the serous surface of the peritoneum, but without organization. 3tio. A liquid exudation, sometimes turbid, sometimes limpid or reddish. More or less of serous and purulent fluid was always found in the abdominal cavity, bathing the surface of the intestines. 4to. M. Broussais also found red clots, sometimes thin, sometimes thick, spread over, in form of membrane, the peritoneum, which was reddened and thickened underneath. Blood itself has been found effused from the peritoneal lining of the abdomen, without any apparent breach of vessel or substance.

In most cases, where acute peritonitis has been cured, and the patient soon afterwards died of other diseases, adhesions were found, similar to those occasioned by pleurisy.

The appearances on dissection, after *chronic* peritonitis, are very different from those above enumerated, as resulting from the acute form of the disease.

In chronic peritonitis the peritoneum acquires a greater degree of morbid thickening, and the inflammation appears to have penetrated to the different structures of the subjacent organs. The effusion is serous, limpid, or greenish, or reddish, with white, purulent-looking filaments floating about. Occasionally we find spread over the whole extent of the peritoneum, or over some of the envelopes which it lends to various organs, a crop of granulations, pisiform, white, and not unlike certain miliary eruptions of the skin. Bayle, who has examined these granulations very minutely, observes that, in a subject who presented them to a great extent, he could scrape them off very easily with the scalpel, in many places, and *there* the peritoneum underneath appeared perfectly sound. In other parts, however, they could not be raised from the membrane without tearing the latter. Broussais has also related numerous cases where the peritoneum was larded, and immensely thickened with crops of tubercles.

"I have also (says the same author) seen a species of vesicles, similar to hydatids, formed of the most limpid serum, under a transparent sheet of membrane which had been elevated thereby."

"J'ai vu des especes de vésicules, semblables a des hydatides formées par un amas de la sérosite la plus limpide, sous un feuillet transparent qu'elle avait soulevé."

Broussais observed that thin men, of a lymphatic temperament, and who had been weakened by any disease, particularly by protracted intermittents, were the most subject to these tuberculated disorganizations of the peritoneum.

The subjects opened by M. Laennec presented the following morbid appearances in the abdomen. On perforating the peritoneum a quantity of gas rushed out, which had the odour of sulfurated hydrogen.* The intestinal canal was found singularly conglomerated, and agglutinated into one mass, and partly covered by thickened and adherent epiploon. In some cases the intestines were entangled and twisted upon each other, and glued together by false membranes. The peritoneum itself was thickened, disorganized, and tuberculated, in the manner already described, with effusions of various kinds in the cavity of the abdomen.†

It is evident that these chronic disorganizations of the peritoneum, cannot long exist without affecting the structure, and consequently the functions of the various abdominal viscera. Hence the digestion, chylification, biliary secretion, &c. all become deranged, and present a complication of distressing phenomena. But not only is the disease propagated to the organs over which the peritoneum is spread—it is not seldom extended to the serous tissues of other cavities than that of the abdomen. And here we shall detail a case which will set in a very clear point of view the superiority of English *practice* in inflammatory diseases, over that of our continental brethren, notwithstanding the light which pathologi-

---

* See the ingenious observations of Dr. A. B. Granville on this subject, in a paper read before the Royal Society, and partly published in Professor Brande's Journal.

† We cannot help lamenting here the exceeding barrenness of Dr. Abercrombie's *pathology* in the disease under consideration. It is given in two or three lines. "On dissection we find uniformly effusion of coagulable lymph; in some cases very extensive, and frequently effusion of a turbid or puriform fluid, sometimes in considerable quantity. Gangrene is rare." 167. The case-book of no *private* practitioner (however ample his range of practice) can furnish sufficient *pathological* details. It is in great civil or military establishments, where dissection is universal and unobstructed, that we are to find minute and comprehensive pathology. This article will exhibit so forcible an illustration of the truth of the above assertion, that we shall say no more on the subject here.

cal anatomy has diffused over the subject in France particularly.

"*Case.* Gothard Lich, aged 25 years, a fusileer in the 2d Regt. of Hessian Infantry, was received into the hospital, at Dantzic, on the 3d of April, 1812. On the 4th he was seen by M. Gasc; his skin was very hot, with intense pain of forehead and occiput; face and eyes red; tongue white; respiration laborious; acute pain in the left side of the thorax; constipation of bowels; urine scanty and high coloured; burning heat in the soles of the feet; pulse strong, frequent, and full. The patient was robust, and had experienced a well-marked shiver the evening before, when mounting guard. M. Gasc prescribed a bleeding from the arm, with a *nitrated decoction of marsh mallows, and emulsions with some cooling powders!* (no purgative was ordered.) Besides the above means, fomentations were applied to the abdomen and chest. The patient experienced a temporary relief; but in the evening an exacerbation came on, and the night was spent in great agitation. (No repetition of bleeding was ordered to meet the rising storm of inflammation! No! one bleeding in one day was thought quite enough!)

"April 5th. Venesection was repeated, and the other medicines continued. The day and succeeding night more calm.

"6th. The fever appearing mitigated, and there being some gastric symptoms present, M. Gasc ventured on the exhibition of a little cream of tartar, which procured two or three motions.

"7th. The patient has had a bad night, with great heat and restlessness; the head painful and heavy; the eyes red and inflamed; the countenance animated; temporal arteries throbbing; tongue dry; skin arid; pulse quick and full. (Who, on this side of the Channel, would not have bled profusely from the arm or temporal artery in this state of things?) Leeches were applied to the temples, with some relief; barley ptisan, with nitre, and oily emulsions, alternating.

"8th. Restless nights; symptoms as high as ever; heat insupportable; mouth dry; stupor; alteration of the features; oppression of the chest; delirium; tension and swelling of the abdomen. *Ptisans and emulsions continued. Cold applications to the head; emollient glysters.*

"9th, Delirious all last night—some remission this morning; yet the pulse is strong, frequent, and irregular, with stupor, dry tongue, and intense thirst; abdomen distended and tympanitic, with tenderness on pressure. Fomentations, nitrated decoctions; and, to please a Brunonian brother officer who was present, *camphor*, *æther*, *valerian*, *&c.!* Towards evening (as might easily be anticipated) an exacerbation, ushered in by a slight rigor.

"10th. Delirium still higher last night than during the preceding, and the patient was constantly putting his hands up to his head, as though that were the chief seat of the complaint; difficulty of breathing; *pulse frequent and strong*. Desisted from the Brunonian stimuli, and applied leeches to the head. The symptoms were alleviated, and the patient passed an easy day and night.

"11th. *Spontaneous* evacuations from the bowels, with relief; but towards evening all the violent symptoms returned."

We have not patience to relate the daily mismanagement of this case. Some calomel and digitalis were given, with temporary relief; but no bleeding, blistering, or purging were ever dreamt of, and this fine young man expired on the 19th, after unheard of sufferings!

"*Dissection.* Much blood in the skull; sinuses gorged; vessels of the pia mater injected; pia mater in some places inflamed, in others, exhibiting traces of real suppuration; not much water in the lateral ventricles, but the fourth ventricle full of serous effusion.

"When the thorax was opened, much bloody serum rushed out; the pleura costalis and pulmonalis of the right side glued together, with portions inflamed and suppurated. Substance of the lungs sound; but the cavity of the pericardium filled with purulent serum. The heart every where covered with a yellow membraniform exudation, two lines in thickness. The internal surface of the pericardium covered with a similar coat. The peritoneum throughout its whole extent, both where it lines the parietes of the abdomen, and is reflected over the various organs, was red, and injected with blood. The cavity of the abdomen itself contained a quantity of whitish purulent serosity, bathing the viscera and peritoneum. Liver enlarged and gorged with blood. The mucous surface of the stomach and bowels perfectly healthy."

The above case should be written in letters of gold, and hung up in every ward for acute diseases, in hospitals and infirmaries; nay, if it were placed in every medical man's study, and glanced over before he went out on his rounds, it would prove an invaluable beacon to guide him clear of Brunonian rocks and quicksands. Such effects of disease, fortunately, could not be seen in this country, where we are not such disciples of Hippocrates, and the "medicine expectante," as to hope for Jupiter to come down and drag our waggon out of the mire, while we ourselves stand by with our arms folded. An English patient told a French physician, that "in his [the Englishman's] country they treated the patients as though they were horses."* The able physician to whom this was said (our esteemed friend Dr. Ducamp) will peruse the above case, and these reflections on it: and we appeal to his judgment and candour, whether his countryman would not have acted more wisely in putting this *equestrian* discipline in force with his patient, rather than treat him as though he were composed of such machinery as would not bear the least interference of art.

* "Dans mon pays, on traite nos malades come des cheveux."

*Journ. Gen. de Med. February* 1820.

We confidently hope that the free intercourse which now subsists between these islands and the continent, will mutually enrich the profession on both sides of the channel. We candidly acknowledge our inferiority in *pathology*, and shall not be ashamed to profit by the researches of our neighbours. Let them evince similar wisdom, by giving the energetic *practice* of British physicians a fair trial; and we prognosticate that their ultra-adoration of Hippocrates will diminish.

### IV. Treatment of Acute Peritonitis.

Here we shall, in a great measure, abandon the writings of our foreign brethren, for very obvious reasons, and confine ourselves to our own country; nay, we shall draw the materials of this section of the article principally from our own observations and experience, which are often serviceable in enabling us to judge between cabinet speculations and clinical deductions—no unimportant function of a reviewer, and in which, some medical critics of the present day evince a plentiful lack of discrimination.

We have taken various opportunities, in this Journal, of drawing the attention of our brethren to the intimate connection between the nervous and vascular systems; or, in other words, between irritation and inflammation. In peritonitis, it is particularly desirable to remove, as much as possible, every source of pain and irritation, by derobing the patient of all articles of clothing which might compress the abdomen, by enjoining quietude, by an easy position in bed, by pure and mild air of the apartment, and by the prohibition of all intrusive and useless visitations. These are necessary precautions, which are too often overlooked by the inexperienced; but which mark the practitioner of science and observation.

If, on examining the abdomen, we find local *pain on pressure*, whether general fever has been lighted up or not, we shall seldom act wrongly, by attributing that pain to inflammation, and by treating it as such. The measures pursued in the phlegmasiæ of this country are few in number, but decisive in effect. Although the efficacy of blood-letting, especially in peritonitis, is proportionate to the earliness of its employment; yet we do not see any good reason why it should not be put in force at any period or stage of the disease, *while inflammation exists.* If inflammation be on the point of terminating in gangrene or effusion, what will prevent these, if blood-letting do not? The patient may not bear the same measure of evacuation at a late as at an early stadium of the complaint, but the indications are precisely the same at both periods; viz. 1mo. To lessen the force of the heart, and the fulness of the general vascular system. 2do. To relieve the vessels of the particular part inflamed, of their overplus of blood and irritation. General and local bleedings are the grand means of effecting these objects; but they are not the sole means.

As we know that peritoneal inflammation may be going on insidiously, without much affection of the general system, we ought rarely to commence in any other way than by venesection; and that carried to the extent of relieving local pain, or inducing syncope, or a strong tendency to it. Where the symptoms are previously equivocal, this measure very often gives a fuller development to the nature of the disease, and seldom, indeed, can any danger be apprehended from it, even where it may not have been essentially necessary. The second measure is *local* detraction of blood, by means of leeches applied in considerable numbers over the abdomen, and, if prejudice will permit, to the hæmorrhoidal or vaginal vessels. It is at the moment that the first ease is experienced from general and local bleeding, that the mucous membrane of the intestinal canal is to be excited to copious secretion and complete detrusion of its contents. Here then we differ, *toto cælo,* from Dr. Abercrombie.

"We have seen no reason (says he) to suppose that the retention of fæculent matter was injurious in the one case, or that the copious evacuation of it was productive of the slightest benefit in the other. On the contrary, there are facts which must lead to a suspicion that the action of purgative medicine upon the intestinal canal, when it is in a state of inflammation, rather tends to increase than diminish the disease."*

Were we called to a man with a fixed pain in the abdomen, constipation of the bowels, gastric irritability, fever, and other symptoms indicative of enteritic inflammation, and *then* prescribe purgative medicines, without the preliminary and preparatory measures of general and local detraction of blood, we should expect the results which Dr. Abercrombie alludes to. But surely, this is a very insulated or oblique view of the subject. For what reason do we employ active purgation in the early stages of *thoracic* inflammation? To lessen the whole mass of circulating fluids, and reduce the general action of the heart and vascular system. That purgation does this, we appeal to clinical experience. Now, in abdominal inflammation, provided the *mucous* tissues are not

---

* Edinburgh Journal, April 1820, p. 179.

inflamed, purgative medicines excite the secreting vessels, not only of the whole internal surface of the intestines themselves, but of the glandular organs whose excretory ducts open into the primæ viæ, and thus powerfully deplete *locally*, the vascular system of the abdominal viscera.

When that portion of peritoneum reflected over the intestines is inflamed, but where the villous coat is unaffected, we hesitate not to assert, from personal experience, that constipation of the bowels will, in nine cases out of ten, be a feature of the disease; and in such cases, we maintain, that to excite the natural action of the mucous membrane, immediately after proper vascular depletion, is a powerful mean of checking the said peritoneal inflammation; in the same way that a free expectoration from the mucous membrane of the lungs relieves the vascular turgescence and inflammation of the parenchymatous structure or pleural covering of the same organ, as every practitioner must have repeatedly remarked. And here we appeal to the experience of our professional brethren, whether they have observed that patients generally complain of aggravation of the symptoms after the operation of purgative medicines, as Dr. A. states? For our own parts, we have generally observed the reverse of this take place, when purgation was effected in the manner, and with the preparation above described.

Dr. Parr, the able author of the London Medical Dictionary, after thirty years' experience, states, that in enteritis

> "The salutary termination is by a discharge of fæces. If this can be obtained, the patient is safe; but unless free, copious, and truly fæculent stools are procured, the most promising appearances, in every other respect, will deceive." *Dict. in verbo inflam. intest.*

It must also be remembered, that inflammation of the peritoneal and muscular coats of the intestines has a natural tendency to produce spasmodic contractions of their calibre, and thus lock up whatever fæcal matters may be contained in the bowels, giving additional cause of irritation. The reduction of this spasmodic contraction by blood-letting, warm fomentations, glysters, or even narcotics, and the subsequent action of purgatives, all tend to relieve pain and check inflammation, as we have seen in numerous instances.

Dr. Abercrombie is neither singular nor original in his anti-purgative doctrines in peritoneal inflammations. Broussais thinks purgatives are only proper towards the close of the acute stage, "because the vermicular contractions which they excite in the intestines, must increase the morbid sensibility of the peritoneum." But knowing the inertness of the vascular depletion among our continental brethren, we cannot attach much importance to their therapeutical precautions. Broussais admits the propriety of gentle anodynes, when the force of the reaction is a little subsided.

The success which has attended the administration of oil of turpentine in puerperal peritonitis, first recommended, we believe, by Dr. Brennan of Dublin, is a proof of the power of purgatives in this disease. The turpentine is not only a potent cathartic, but excites powerfully the whole mucous membrane of the intestines, and thus derives the morbid irritation from the peritoneal tunic to a secreting surface, where it is carried off by the increase of secretion itself. Oil of turpentine and castor oil form a very useful combination for exhibition in this disease.

By these observations in favour of purgative medicines, in acute peritonitis, we do not mean to lessen the absolute necessity for decisive vascular depletion, general and local, previously to their administration. On the contrary, we urge the propriety and priority of these means, recommending the use of purgatives as an auxiliary measure. Our usual practice, indeed, in this dangerous disease, is, first, to order general blood-letting till the pain is relieved, on pressure, or syncope produced; and while the local detraction of blood, by numerous leeches, is going forward, we order a grain or two of opium, or six or eight of extr. hyoscyami, combined with five or ten of calomel, and six or eight of ex. col. comp. to be taken. After the local bleeding, and a couple of hours after the above medicine has been swallowed, we endeavour to set the bowels in action, either by neutral salts, castor oil, or laxative injections. Reiterated observation has proved to us the utility of combining an anodyne with the first cathartic, and especially with the calomel, as it tends to prevent or allay gastric irritability, intestinal spasm, and irritation, thereby insuring a more copious fæcal evacuation afterwards, than would otherwise be procured.

We observe in Dr. Gregory's MS. Lectures, the following passage, which is sufficiently in point.

> "Small doses of the sal. glauberi are better than the common purgatives; but as salts seldom sit easy on the stomach in this inflammation, it is necessary to join an opiate, *which does not stop the operation of the purgative, as one would imagine,* but only keeps the patient from vomiting." *MS. Lect.*

Dr. Pemberton's sentiments respecting purgatives in enteritis, general and local blood-letting being premised, are as follow:

> "If the stomach will bear liquids of any sort, a strong solution of magnes. sulph. in aq. menth. pip. with an addition of tinct. sennæ, may be directed in such quantities, and at such intervals, as the sickness of the stomach will allow. If, however, all liquids are rejected, we may direct an usual dose of calomel, in union with the ex. col. comp. every six hours, ad quartam vicem. In the intermediate hours, an injection of water-gruel, with common salt, may be employed. Purgatives are to be continued during the whole progress of the disease." *Fourth Edit. p.* 181.

It is very strange that this able and experienced physician should not have noticed what Dr. Abercrombie considers as a common occurrence in enteritis—a natural state of the bowels, and aggravation of the complaint by purgatives. We believe, however, that Dr. Pemberton is not very singular in this respect. Dr. Abercrombie appears to be fast falling into the error of converting exceptions into general rules.

We agree with Dr. Abercrombie, that the first blood-letting should be as early as possible, and carried the length of making a decided impression on the system; and also that, to prevent this advantage being lost, we should repeat the depletion, after a short interval, whenever the effect of the preceding one has subsided. If the venesection be thus *timeously* repeated, it will seldom require a large detraction to check the pain and inflammation; whereas, if the disease be allowed to acquire force in the intervals of depletion, it will not only require a large loss of blood, but that large loss itself will have less chance of bridling the inflammation than the timely small one, while it seriously reduces the vital energy of the patient. And this is an additional reason for keeping up a secreting action on the mucous membrane of the bowels, which saves the detraction of pounds of blood.

Dr. Abercrombie wisely cautions the practitioner not to lose sight of a patient with an inflamed vital organ more than an hour or two at a time, until the force of the disease be decidedly broken. Unless the violence of an internal inflammation be signally mitigated within the first twenty-four hours of active treatment, the eventual result will be very doubtful.

Dr. Abercrombie, and indeed the practitioners of this country in general, employ local blood-letting in abdominal inflammation with too sparing a hand. Not only should leeches, in great numbers, be applied over the abdomen, but to the anal and vaginal regions, the bites being encouraged to bleed as long as possible, by warm fomentations.*

* An able friend of ours, in the case of a very delicate female child, seven and a half years old, had lately fifty-five leeches applied to the abdomen, in the course of a very few days. The pulse, before the leeches were applied, could hardly be counted. After the first application of fif-

If conveniences are at hand, the warm bath should succeed general and local blood-letting, not only as a means of equalizing the circulation, but of lessening pain, and promoting the effect of the purgatives. If the warm bath cannot be procured, reiterated fomentations, with decoction of poppy heads, chamomile flowers, and a proportion of spirit, should be applied to the abdomen, which will not interfere with the bleeding from the leech-bites. Broussais recommends, in addition to the tepid bath and fomentations, gentle friction over the limbs, with the hand or some other body supple and agreeable to the touch.*

After the violence of the inflammatory action is subdued by general and local blood-letting, laxatives, and fomentations, or the warm bath—*blisters* are important auxiliaries. Broussais recommends the first blisters to be applied to the thighs or legs, in order to keep the surface of the abdomen clear for leeches and fomentations; but when the acute stage is declining, or if the other measures seem to fail, then we are to cover the abdomen with a blister. We ourselves have not hesitated to blister the abdomen, as soon as a decided impression was made on the disease, by the other and more essential measures. Leeches, if subsequently necessary, may

---

teen leeches, it fell to 140, with frequent intermissions, both then and subsequently. When the violence of the disease was broken, the pulse remained five days, viz. from the sixth till the eleventh of April, stationary at 112. At this time, an ounce of castor oil produced several motions, in which were some olive-coloured lumps that appeared to have been long retained. The pulse now fell below 100, for the first time since her illness, " evidently showing (says he) how important a free state of the bowels is; for the pulse has risen or fallen, according as the bowels were confined or free."

* Dr. Hall, of Berwick, in a Letter to Dr. Dickson of Clifton, on the subject of peritoneal inflammation, observes, " in all active inflammations of this kind, the suddenly emptying the blood-vessels, generally affords such relief at first, as to give time for topical applications being afterwards made with advantage. I have found benefit from small doses of ipecacuan joined with calomel, where the alvine discharge is slow, provided the stomach is not in an irritable state; in which case, the addition of ol. ricini to a common enema, often answers the best purpose."

Dr. Dickson himself, in a letter to the writer of this article, observes, " I think it of the highest import, towards conducting the disease to a safe termination, to keep the bowels freely open by mild means, and by frequent injections; for I have repeatedly remarked the pulse to fall after a copious motion." He justly advises, of course, that the force of the inflammation be previously broken by decisive blood letting, as we have already inculcated.

Dr. Pemberton, in his valuable work, states, " it is necessary that the bowels should be kept *constantly* open." He recommends castor oil or neutral salts.

be applied, with great advantage, to the flanks of the abdomen, and to the anus.

Of the cold applications recommended by Dr. Abercrombie, Dr. Kinglake, and several French writers, we have not any experience.

"In a considerable number of cases (says Dr. A.) I have used, with evident advantage, the application of cold, by covering the abdomen with cloths wet in cold water or iced water, or by pounded ice in a large bladder."

The authorities for tobacco injection, in inflammation of the bowels and ileus, are exceedingly numerous, as the reader will perceive, by looking into Plouquet, (vol. v. p. 117) where, among others, De Haen and Fowler have recommended the measure. Without much experience in this remedy ourselves, we are disposed to unite with Dr. Abercrombie, in proposing to revive its use as a powerful auxiliary in the disease under consideration.

"The great advantage (says Dr. A.) attending the use of it in enteritis is, that while it tends to move the bowels, and keep them free from distention, it is, at the same time, powerfully calculated [he ought to have said, "calculated powerfully"] to allay vascular action, and may thus assist in keeping down the inflammation."

Mr. Howship, in his late work, mentions his having seen three cases, "in which the patients had suffered extreme pain in the region of the bowels, and in which the prescriptions of physicians had availed nothing. Mr. Heaviside being consulted, and finding that the stomach had rejected every thing taken, and that stimulating glysters produced no favourable effect, in despite of bleeding, warm bath, and almost every other means, desired Mr. Howship to try the fume of tobacco, injected cautiously, by the proper apparatus. A general commotion and rumbling noise in the bowels took place in all these cases, and were soon followed by copious evacuations of fæcal matter. The patients were saved."—*Practical Observations, &c. p.* 19.

Of the sedative effects of antimony on the vascular system, we dare not avail ourselves in abdominal inflammations, on account of the gastric irritability; but Dr. Abercrombie recommends the free use of digitalis, in cases where the pulse continues frequent after the local symptoms appear considerably subdued by bleeding, and consequently where there is danger of relapse.

A tympanitic state of the abdomen is certainly a formidable symptom in enteritis, and a very unfavourable omen. We have seen it, however, owing to a sudden extrication of gaseous fluid within the intestines, and removed by aromatics, friction, and assafœtida injections. Dr. Abercrombie has seen injections, with large quantities of cinchona, very useful.

There is a period, in some cases of abdominal inflammation, where the disease is just subdued, but where there is a kind of balance between recovery and gangrene. The pain will vanish, the pulse become weak, the vital powers appear to sink, and a coldness overspread the body. These symptoms are too often indicative of mortification; but every experienced practitioner must have occasionally witnessed cases of recovery, even from this alarming state. Here we must give wine; for if gangrene *have* commenced, no harm can ensue from the remedy; and if it have *not* commenced, the wine may happen to give a salutary stimulus to the nervous and vascular systems, when stagnation is on the point of taking place in the vital fluid, and where farther evacuations would be instant death. In some cases of this kind, however, the symptoms are so equivocal, as to render it exceedingly difficult to determine on the proper course to pursue.

"A lady, (says Dr. Abercrombie) aged about 35, on the seventh day after delivery, was seized with violent pain over the whole abdomen, most severe across the stomach and towards the right side; much tenderness on pressure; urgent vomiting; great restlessness; respiration short and oppressed; pulse 140, and sharp. The pain was aggravated by inspiration, and by every motion of the body. She was bled and blistered, and took laxative medicine, which operated freely. After the bleeding she was very much relieved; could breathe without uneasiness; the vomiting subsided, and the pulse was much diminished in frequency: this was in the night. On the following day the pulse rose to 150; the breathing was quick, short, and oppressed; some vomiting; countenance anxious; there was neither pain nor tenderness of the abdomen, which was to the feel soft and natural; lochia natural. Wine was given in the quantity of a small glass every hour, and injections of beef tea, each containing ℥ß of bark in powder, and sixty drops of laudanum. These were repeated as often as they were discharged, which was generally from one to two hours. After some hours the symptoms were improved. Next day the pulse was from 125 to 130; on the third day from 112 to 120. Thus she gradually recovered, having continued to take a bottle of wine in each twenty-four hours." *Ed. Journal, April,* 1820.

It is hardly necessary for us to observe, that the strictest antiphlogistic regimen is essential throughout acute peritoneal inflammation. Saline draughts, in a state of effervescence, will be useful in allaying thirst and gastric irritability—and when food is necessary, the farinaceæ should long precede animal food, or any other kind of ingestion which produces much fœcal remains. Rice water for drink is very useful in convalescence, as it is nutritious but not stimulant; and as this disease leaves a strong tendency to relapse, the patient should be enjoined the strictest attention to abstemiousness, regular bowels, warm feet, and flannel next the skin, for a long time after every symptom has disappeared.

*Treatment of Chronic Peritonitis.* In the first edition of M. Broussais's work, he appears to consider chronic peritonitis as incurable; and M. Gasc, the writer of the article peritonitis, in the Dictionnaire des Sciences Medicales, gives a similar prognosis—" on peut dire que la péritonite chronique est presque toujours mortelle." Within the last twelve or fourteen years, however, M. Broussais has met with several cases of this disease which terminated favourably. He justly observes, nevertheless, that in many instances, chronic peritonitis is supposed to be cured when the disease is merely dormant for a time, and pretty sure to advance again with hasty strides. M. Broussais gives a satisfactory explanation of the general incurability of the disease; namely, that tuberculous and pultaceous matter is commonly deposited, during chronic inflammation of the peritoneum, between the layers of this membrane, or in the substance of the false membranes thrown out on its internal surface, which depositions appear to be incapable of absorption; and, consequently, keep up an irritation which feeds the disease. This tuberculous matter may, for a time, lie dormant, or create little inconvenience: but ultimately, it kindles up irritation and inflammation. It is difficult, however, to know the exact period when these extraneous matters are deposited, and therefore we should not be too abrupt in giving an unfavourable prognosis. M. Broussais thinks, that a patient's fate, in general, depends on the treatment during the first twenty or thirty days of the disease, though he does not deny that it is curable at a much later period. Indeed, where chronic peritonitis steals on, without having been preceded by the acute form, it is seldom we see the complaint during the period above mentioned.

In respect to the treatment, our first object is to ascertain if there be any remains of the acute or sub-acute forms of the inflammation; for, in that case, we must still employ a modification of remedies already described. And, indeed, where no acute peritonitis has preceded, we are to be ever on the watch for a supervention of such a state; and ready to combat it by active means.

Our next object is to lessen or soothe irritation and pain, which aggravate the disease and accelerate its march. The food should be of the very lightest kind, so as that but few fæces may be formed, and those should be daily carried off by the gentlest means, particularly by laxative glysters. It is evident that when coagulable lymph has been thrown out, and adhesions have taken place between the intestines themselves, or between them and any other parts, the action of purgatives must be productive of serious mischief. The case is here totally different from acute inflammation of the peritoneum, and before any disorganizations have taken place.

In this chronic or apyrectic peritonitis, we must endeavour to arrest the morbid process going forward internally, through the medium of the skin. The tepid bath, fomentations, stimulating frictions, blisters, or even issues, should be successively employed, with or without leeches, according to the activity or inactivity of the internal inflammation.

As a serous effusion is a common consequence of this dangerous disease, and is ever to be dreaded, *diuretics* are important remedies, particularly the digitalis, united with some gentle saline aperient, as the acetate of potass, or soda tartarizata.

The following will be found a very powerful diuretic in many cases besides that immediately under consideration.

℞. Acidi tartarici . . . . . . . ℈j.

Sodæ carb. . . . . . . . gr. xxiv.

Infusi digitalis . . . . . fl. ℥ß.

Spir. ætheris nitrici . . fl. ʒj.

Tinct. scillæ . . . . . . ♏. iv.

Aquæ menthæ . . . . . . . . ℥ij. M. ft.

Haustus bis terve in die sumendus.

Broussais strongly recommends the introduction of diuretic medicines, as the tinct. lyttæ, and tinct. scillæ, by means of friction on the skin, aided by gentle laxatives internally. It is needless to observe, that the most perfect quietude is necessary in this complaint; perhaps, however, a sea voyage, from its known effects on the absorbent system, and a change to a warm climate, might be useful here. Anodynes must be exhibited; but they should be such as produce the least constipation, as hyoscyamus, conium, &c. If opium be used, it must be combined with laxatives. Without asserting that the disease under consideration is *identical* with what Dr. Baron has described, under the term "tuberculated accretions of the serous membranes," in his valuable work on that subject, we may at least be permitted to consider the two affections as very *analogous;* and under this idea, the plan of exciting to absorption by nauseating medicines, as recommended by the illustrious Dr. Jenner, in the work alluded to,* is highly deserving of a trial, in so intractable a disease. There can be little doubt, indeed, that the absorbents might be made to act upon many extraneous and morbid growths in the human body, by *rigid abstinence* alone, if patients had fortitude to persevere in the measure. For our own parts, we should place more confidence in this than in any other remedy; and practitioners should at all times bear in mind, that without strict abstemiousness, there is little hope of a cure in chronic peritonitis.

We shall now introduce a few examples of the acute and chronic forms of the disease under review, from M. Broussais, which will afford ample food for reflection to our readers, and give us an opportunity of making some incidental remarks of our own.

*Case. Acute Peritonitis simulating low Fever.*

"A man, 26 years of age, large stature, and robust, entered the hospital of Medenblick on the 4th fructidor, with an air of great suffering, livor of countenance, sharpness of the features, parched tongue, delirium, constant jactitation. He complained of nothing; but the abdomen was tender on pressure, pulse quick, depressed, and feeble. These being the features of low (ataxique) fever in the last degree, *I could only prescribe tonics and antispasmodics* for the night.

"Next day, (10th of the disease) about one o'clock, the abdomen became intolerably painful, and the patient could not bear the slightest pressure without making great complaint. *A lavement, emollient fomentations.* No relief. The patient got into a convulsive agitation, uttering piercing cries. The warm bath for three quarters of an hour, in which time several spoonfuls of an antispasmodic potion, with laudanum and ether. After coming out of the bath he was easy and tranquil, the pulse fuller, and the skin soft. He could now give some account of his complaint. In the vessel on which he embarked at the Texel, he became affected with some gastric symptoms, followed by nausea, cold chills, and malaise. A vomit was given him, during the operation of which, he first felt the pain in the abdomen, which had never since ceased. I now perceived that the disease was peritonitis, and prescribed *leeches, fomentations,* and *diluents.* The evening was calm, and only a little sensibility on pressure of the abdomen remained.

"Next day, (11th of the disease) the abdominal pains renewed, with restlessness. *Fomentations.* The warm bath was attempted, but the pain was so great that he could not be put into it. Constant tossing about during the remainder of the evening, with exquisite

* See also our *Quarterly Series,* No. 6, for October 1819.

tenderness of the abdomen. I was deterred from again leeching the abdomen, by the sinking of the countenance, and weakness of the pulse; indeed, I judged that the process of disorganization had commenced. *Anodynes.*

"The day following, all was calm, and he said he was 'very well;' but he died in the evening.

"*Dissection.* Corpse extremely muscular. Vessels of the head a little injected, with some serous effusion. Peritoneum red, covered with sanguiferous vessels, and thickened to a line or a line and a half, particularly over the intestinum ileum, where there were several black spots. The peritoneal covering of the mesentery, omentum, and ileum, was coated with a solid exudation of a whitish yellow colour, strongly adherent. The muscular and mucous coats of the intestines sound."

M. Broussais observes, that at the period when this patient arrived under his care, no measures would probably have saved him. We do not agree with him. We are sure that the *symptoms* authorized *general* blood-letting as well as local; together with intestinal evacuations; and we are disposed to think, that under the energetic treatment of this country, the patient might have been saved. At all events, the methodus medendi was here as bad as it possibly could be, and dissection evinced the inefficacy of nature, or the "medicine expectante," in counteracting peritoneal inflammation.

We agree with M. Broussais, indeed, that in the above case, the emetic was injudicious, nay injurious; and well may our author exclaim, "Si les nausées qui décidèrent l'emploi du vomitif dépendaient d'un principe de péritonite, combien le malade est à plaindre qu'on n'ait pas les interpreter!" *Vol. II. p.* 408.

The following case is well worthy of every practitioner's attention.

*Case. Acute Peritonitis simulating Spasmodic Colic.*

"Bongeot, ætat. 39, robust and athletic, entered the hospital of Udina, the 7th of August 1807, for the treatment of violent colic, with which he had been tormented during nine preceding days. He had a dull but constant pain in the abdomen, exasperated every night, and for which he had taken, without relief, spices, wine, lavements, and various stimulants. He was affected frequently, during the above period, with vomiting, and the constipation was invincible.

"He had now a countenance indicative of suffering, a flush on the face, contracted pulse, no way frequent, and rather feeble than strong, with little heat of skin, no abdominal distention, and but little tenderness on pressure. I prescribed 'la solution Gommeuse acidulée et des juleps anodyns,' with some amelioration of the symptoms.

" 8th. This day passed with some little malaise, a sense of painful fulness in the belly, and no appetite. Same treatment.

" 9th. The obstinacy of the constipation appeared to demand some evacuants. Tamarind decoction. Great increase of pain, and restlessness, but no fever. No stool. *No medicine, but mucilaginous drink.*"

The complaint went on gradually increasing for some days, with a developement of fever, and at length M. Broussais opened his eyes, and saw that he had peritonitis to deal with! *Fomentations, six leeches to the margin of the anus.* Great reduction of the symptoms, and tranquil night. But the next morning brought a renewal of all the symptoms, with fixed and constant pain in the abdomen, which was increased every night to horrible tortures. Emollients; repetition of the leeches to the anus and lower part of the belly. In short, he dragged out a wretched existence till the 19th of August, without the least *active* measure being taken by M. Broussais, the most *active* and energetic physician in all France!

" *Dissection.* The corpse very fleshy and muscular; all the muscles in a state of rigid contraction. Chest sound, *Peritoneum* red, and thickened throughout its whole extent, and covered, in some places, with a whitish exudation. There was a small quantity of serious effusion in the abdomen. The mucous membrane of the *stomach* appeared red; but that of the intestines was perfectly healthy." 412.

It is really too bad to hear M. Broussais throw the blame here upon the *tamarind decoction!* " on la voit, d'abord obscure s'accroître prodigieusement *par l'effet d'un purgative." (!)* M. Broussais even outstrips Dr. Abercrombie in his condemnation of purgatives in peritoneal inflammation. " Les purgatifs seraient presque aussi pernicieux que les vomitifs, dans cette maladie." But we beg Dr. Broussais's pardon when we say, (and indeed without taking much credit to ourselves) that we would have saved Bongeot's life, by bleeding and *purging*. These cases, however, are invaluable to the profession. They teach us what may be *done*, by exhibiting what has been left *undone*.

The above case is valuable in another point of view. It proves to us that acute inflammation of the peritoneum may exist in a very high degree, and accompanied by great pain, without any febrile movement in the general vascular system.

M. Broussais here relates an interesting case of what he denominates " acute hæmorrhagic peritonitis." A soldier 28 years of age, athletic, and a great eater, had, for some time, been subject to attacks of pulmonic inflammation, with slight hæmoptyses. About the middle of September, 1800, he became affected with some general febrile symptoms, for which the surgeon of the place prescribed an emetic, as he considered it "la fievre du pays." During the operation of the medicine, the patient experienced a very violent and deep-seated pain in the left hypochondre, after which the fever became strongly developed.—On the 17th he took a purgative, and on the 18th was calm, and sent to the hospital of Udina; where, being seen in the evening by a physician, he appeared in such a state of debility and exhaustion, that antispasmodics and light nourishment were ordered.—19th. A deceitful calm; weakness; cold chills.—20th. Calm in the forenoon; but towards evening, violent fever, great anxiety, severe pain in the side, spreading all over the abdomen, respiration laborious, cold sweats. Death in the night.

" *Dissection*. Nothing remarkable in the head or thorax. The cavity of the peritoneum filled with clots of blood, which were spread in sheets over the principal viscera. Spleen vastly distended with blood. On a more accurate investigation, the cellular tissues beneath the peritoneum, and between its duplicatures, were black, and injected with blood."

### *Case of Chronic Peritonitis*.

" A young soldier entered the hospital at Nimeguen for an affection of the testicle. Some time after his arrival he experienced a gastric affection, which induced the surgeon to order him an emetic; during the operation of which he felt pains in the abdomen, that continued in spite of various sedatives. To these were added, vomiting, dysury, and fever; on which account he was turned over to the physician's wards, where M. Broussais found him, two months after the commencement of the complaint.

" He was now wasted, pale, and affected with cough, unaccompanied by expectoration. The abdomen was distended, and painful to the touch; the patient also experienced constant cutting pains in the belly. Vomiting of every thing taken, and aggravation of all the symptoms after eating any thing of an irritating nature; difficulty of making water; pulse frequent, hard, and contracted; exacerbation of heat, fever, and pains in the evening. M. Broussais pronounced the disease to be incurable, and merely prescribed diluent and anodyne drinks. He died about three weeks afterwards, in an advanced stage of marasmus.

" *Dissection*. Some adhesions in the chest; but the substance of the lungs sound. The abdomen contained a considerable quantity of milky serum. The peritoneum was red, granulated, and, in many places, four lines in thickness. Over its surface were scattered small fragments of a whitish exudation, unorganized, and, in a great measure, dissolved in the abdominal effusion. The liver was enlarged, and resembled granite when cut into, interspersed with tubercles. The mucous membrane of the intestinal canal and stomach perfectly sound." *Vol. ii. p.* 435.

The following case will shew us, that chronic peritonitis may be going on for some time without much derangement of function in the digestive organs.

" *Case.* Nomin, a soldier, 27 years of age, formerly stout and muscular, entered the hospital of Udina, on the 23d of January, 1807, in a state of advanced marasmus, with pain and tumefaction of the abdomen; which, together with the whole surface of the chest, was morbidly sensible to the touch. Face expressive of great sufferings; constant cough, with expectoration; pulse frequent, sharp, and not strong. He stated, that four months previously, he had been attacked by a quotidian ague, during the first eight days of which, he became much swelled about the body, which he attributed to the quantity of water he had drunk in the hot fits. He was treated at the hosspital of Treviso by a continued exhibition of bitter wine; and at the expiration of two months and three days he was discharged, apparently cured. But a fortnight after he left the hospital, he became affected with a pain in the side, about the region of the spleen, accompanied by a diarrhœa. This diarrhœa continued obstinate, with fever, emaciation, and cough; and in about a fortnight from this relapse he died.

" *Dissection.* The pleura pulmonalis and p. cost. slightly adherent by means of a whitish unorganized exudation, and about a pint of serous effusion in the left cavity of the chest. Parenchymatous structure of the lungs sound. The peritoneum a little thickened, and lined throughout with the gelatino-albuminous exudation above-described in the thorax. The whole of the abdominal viscera, without exception, were covered with this production, which served to glue them slightly together. No serous effusion. The mucous membrane of the stomach was a little red, and some reddish patches were observable on the mucous surface of the small intestines, and also of the colon, but without any ulceration." 437.

In the following case, we imagine, Dr. Baron will recognize an instance of the disease on which he has lately written. It presents also a complication of peritoneal inflammation, with phlogosis of the mucous membrane of the intestines.

" Pierrott, 22 years of age, perceived, about the middle of July 1806, a swelling of the abdomen, succeeded by much flatulence, and by diarrhœa, together with pain. These continued a month, when he was obliged to quit duty and enter the hospital of Udina, where he had twenty or thirty stools per diem. The diarrhœa was mitigated for a short time by ipecacuan; but it returned again, and now the patient came under the care of M. Broussais. The patient, at this time, had frequency of pulse, heat, diarrhœa with tenesmus, obscure but permanent pains in the abdomen, with flatulent distention, tenderness on pressure, in the line of the ascending colon.

Rice water, demulcents, &c. with anodynes, and rigidly low diet. By these means the diarrhœa was checked, and the febrile heat reduced in a month; but whenever the nourishment was increased, or tonics given to recruit the strength, then the fever, bowel complaint, and pains of the abdomen returned. The low regimen was therefore again persisted in for six weeks longer; but in spite of all this, there continued a dull pain in the abdomen, especially when *lateral* pressure was applied; and the abdomen was evidently more protuberant than it ought to be. Tired of the hospital, he was sent to his corps (exempted from duty) from the 15th of November till the 5th of January, when he returned to M. Broussais in a state of advanced emaciation—the skin of an earthy colour, the belly somewhat prominent and hard, the pulse very frequent, but not much febrile heat. He said he felt painful twistings in the abdomen, with a sensation as though some globular body was ascending to his throat. He was still affected with diarrhœa. Death put an end to his sufferings on the 12th of January.

" *Dissection.* Excepting some old adhesions and a few incipient tubercles, there was nothing particular in the lungs. *Abdomen.* All the viscera bound together by the disease of the peritoneum, which membrane was thickened, discoloured, and formed, with the diseased epiploon, a dense covering, through which were scattered a multitude of tubercles, filled with whitish matter. On the intestinal peritoneum these granulations resembled variolous pustules. The mesentery was morbidly thickened, and its glands tuberculous.

" The mucous membrane of the stomach was a little red, but only in detached patches; the internal surface of the small intestines little affected; that of the colon was generally red, with ulcerations of greater or less extent. The liver sound, the spleen somewhat tuberculated. On the surface of the diaphragm several tubercular productions as large as a pullet's egg." 450.

We suspect that many cases of what is called *common ascites* would, upon minute investigation, present phenomena analogous to the following.

*Case. Chronic Peritonitis, with Dropsical Effusion.*

" Boulard, a fusileer in the 35th Regt. a man about 30 years of age, stout and muscular, having got cold, by exposure to rain at the siege of Ulm, became suddenly leucophlegmatic. The dropsical effusions, however, gave him so little uneasiness, that he kept at his duty during the winter campaign in the mountains of Styria and Carinthia. In March, 1806, four months after the exposure above mentioned, and the commencement of his complaint, the dropsy forced him to enter the hospital of Udina.

" He had now general anasarca; complained of no particular pain; had only a little malaise and dyspnœa, with some cough at night. Pressure on the abdomen caused no pain, unless it was very strongly made, and then it was so obscure and confused that no indication could be drawn from it, since strong pressure on the abdominal viscera gives uneasiness at all times, even in health. The patient had no fever, an excellent appetite, the complexion unaffected, and, in short, there was reason to hope that the disease might be idiopathic dropsy. By a proper employment of aperient and diuretic medicine, the dropsical effusions were almost completely evacuated in three weeks; but the medicines gradually lost their effects, the dropsy returned, and the patient finally sunk on the 6th of April, 1806, about five months from the origin of his ailments.

" *Dissection.* The lungs much pressed upon by the protuberant diaphragm, and somewhat engorged; but no disorganization of structure. *Abdomen* filled with a whitish serum; the peritoneum thickened, non-transparent, and almost every where lined with a white, pulpy, and easily separable exudation. The mucous membrane of the intestinal canal sound throughout."

The above state of the peritoneum is doubtless the cause, in numerous instances, of those obstinate relapses which occur, from time to time, in cases of ascites. But dissection is too little attended to in death from dropsy. An important practical indication, however, may be deduced from these researches; namely, that where no abdominal viscus appears obstructed in ascites, we should examine minutely into the state of the peritoneum, and *generally* suspect some inflammatory action in that extensive membrane. Much might probably be done by leeching the abdomen, and blistering, while evacuating the collected effusions by diuretics and aperients.*

We shall extract but one more case of this disease, and may it prove a useful beacon against the dangerous doctrines of *debility*, on which even a Broussais has too often been wrecked!

" *Case.* Pagnet, 22 years of age, a fusileer, in the 84th Regt. received a wound in the foot; for the cure of which he entered the hospital of Udina, and remained there three months, with the following symptoms. From the time of his entrance he complained of pain in the belly, which was very tense, while the aspect of his countenance evinced that he had been unwell for some time. The wound of his foot would not heal, but remained stationary in spite of every thing they could do. He had evening fever. Tonics and stimulants always made him worse, and frequently nauseated his stomach. About the middle of May, fifteen days before his death, Pagnet complained that the pains in the abdomen, which had hitherto been of a dull obtuse kind, were now become acute; and, in a short time, they became so aggravated that the weight of the bed-

* See our Review of Dr. Crampton's excellent paper on dropsy, in No. 5, Quarterly Series.

clothes was insupportable. Fever was now strongly developed, with burning heat, and much fœtor of all the excretions. The stomach would not retain any thing, and he was harrassed to the moment of his dissolution, with the most excruciating sufferings and ardent fever. '*Il souffrait continuellement des doulours horribles, et etait devoré par une fièvre ardente qui, par sa violence, paraissait fort au dessus des ses forces*'."

It does not appear that any depletory measures were put in force, at any period of this wretched man's complaint! The following ravages of inflammation are coolly detailed, without M. Broussais's appearing to even dream that there was the least mis-management of the case!

"*Dissection.* Evidence of universal peritonitis, with a concrete exudation, while a black, purulent sanies, of stercoraceous fœtor, filled all the intervals which the adhesions had left. The intestines were sphacelated in numerous points, and perforated like a sieve in many places. Through these perforations, fæcal matter and intestinal gases had passed into the cavity of the abdomen. The mucous membrane of the bowels was sound throughout, excepting at the places where the perforations were situated." 482.

It is totally inexplicable how our continental brethren can see these things, and accurately describe them, without, in the smallest degree, profiting by the results of their pathological labours! If they *will* continue blind to the light of truth, let us profit by their oversights. These pathological specimens could not occur in Great Britain; but they are most invaluable, as establishing the solid basis on which the energetic practice of this country, in acute diseases, rests.

It is now time for us to close this article. We have endeavoured to form a focus, whereby the rays of light, from various quarters, might be made to converge on a single point, and illuminate an important subject. Should our labours meet the approbation of the profession, we shall continue them, while health and life are spared, until this Journal shall become a depositary of medical illustrations that may render it a useful and authentic source of reference, on all the leading points of pathology and practice. If TIME should confirm these (we trust) laudable anticipations, it will hereafter cheer the gloom of ebbing life, to reflect that we have not lived in vain, nor suffered the vigour of manhood to pass without contributing our quota to the mighty impulse which guides the march of human knowledge at the beginning of the nineteenth century.

