# Errata

**Published:** 1820-09-01

**Authors:** 


ERRATA.

During the Editor's absence from England, while a portion of this number was passing the press, some literal errors crept in; which, however, the reader will, in most cases, easily detect. The following are requested to be corrected with the pen.

Page 186, line from bottom 18, for Aq. Menth. *one drachm,* read *one ounce.*

